# Preliminary Study of Quercetin Affecting the Hypothalamic-Pituitary-Gonadal Axis on Rat Endometriosis Model

**DOI:** 10.1155/2014/781684

**Published:** 2014-10-28

**Authors:** Yang Cao, Meng-fei Zhuang, Ying Yang, Shu-wu Xie, Jin-gang Cui, Lin Cao, Ting-ting Zhang, Yan Zhu

**Affiliations:** ^1^Department of Obstetrics and Gynecology, Yueyang Hospital of Integrated Traditional Chinese and Western Medicine, Shanghai University of Traditional Chinese Medicine, Shanghai 200437, China; ^2^Department of Traditional Chinese Medicine, The First People's Hospital of Yinchuan, Ningxia 750001, China; ^3^Department of Reproductive Pharmacology, NPFPC Key Laboratory of Contraceptives and Devices, Shanghai Institute of Planned Parenthood Research, Shanghai 200032, China

## Abstract

In this study, the endometriosis rats model was randomly divided into 6 groups: model control group, ovariectomized group, Gestrinone group, and quercetin high/medium/low dose group. Rats were killed after 3 weeks of administration. The expression levels of serum FSH and LH were detected by ELISA. The localizations and quantities of ER*α*, ER*β*, and PR were detected by immunohistochemistry and western blot. The results showed that the mechanism of quercetin inhibiting the growth of ectopic endometrium on rat endometriosis model may be through the decreasing of serum FSH and LH levels and then reducing local estrogen content to make the ectopic endometrium atrophy. Quercetin can decrease the expression of ER*α*, ER*β*, and PR in hypothalamus, pituitary, and endometrium, thereby inhibiting estrogen and progesterone binding to their receptors to play the role of antiestrogen and progesterone.

## 1. Introduction

Endometriosis (EMs) is a chronic inflammatory disease, characterized by implantation and growth of endometrial tissue outside the uterine cavity. It can influence pelvic environment and immune function, ovarian tissue, and follicular growth, leading to the occurrence of dysmenorrheal, infertility, menstrual disorder, and so on. This disabling condition is considered one of the most frequent diseases in gynecology, affecting 15–20% of women in their reproductive life. The prevalence within infertile women is very high, reaching almost 50% [1]. The pathogenesis of EMs is related to immune factors, genetic factors, endocrine factors, and so on [2].

Although endometriosis has been described since the 1800s, the mechanisms responsible for its pathogenesis and progression remain poorly understood. It is well established that endometriosis grows and regresses in an estrogen­dependent fashion [3]. Therefore, estrogen and its receptors play an important role in the occurrence of EMs; they are necessary for the implantation and growth of ectopic endometrium. Loss of sex hormone balance inside body or the elevation of hormone receptor can both contribute to the occurrence and development of EMs. At present, the treatment of western medicine to this disease is mainly composed of pseudo menopause therapy, and pseudocyesis therapy is the most widely used [4]. But the side effects of these treatments and their long-term effects need further research. Research shows that the nervous system plays an important role in the regulation of the menstrual cycle [5]. Therefore, to find a treatment that can inhibit the growth of ectopic endometrium and does not affect the menstrual cycle at the same time is the ideal method for the treatment of EMs.

Recently, there have been many researches about quercetin, and most of them are related with estrogen correlative diseases, such as breast cancer, osteoporosis, cervical cancer, and so forth [6–8]. Latest studies show that quercetin can inhibit the growth of ectopic endometrium on rat EMs model [9]. This is also confirmed in our recent study. Currently both chinese and other countries researchers still have not deeply studied the mechanism of quercetin on endometriosis treatment. Therefore, our research will explore the mechanism of quercetin effect on endometriosis through three levels of the hypothalamic-pituitary-gonadal axis, especially two target organs of hypothalamus and pituitary and provide the basis for perfecting the pharmacological mechanism of quercetin. Then, we will further deepen the research and reveal the target and internal mechanism of quercetin inhibiting the growth of ectopic endometrium, and provide experiment science basis on the mechanism of quercetin preventing and treating EMs and its recurrence.

## 2. Materials and Methods

### 2.1. Animals

Female Sprague-Dawley (SD) rats (6-7 weeks old, shanghai B&K Universal Group Limited, China), weighing 180–200 g each, were used in the experiments. They were kept under specific pathogen-free conditions with air conditioning and a 12 h light/dark cycle. Rat had free access to food and water during experiments. This study was approved by the Animal Care and Use Committee of Shanghai Institute of Planned Parenthood Research (number 2010-01).

### 2.2. Chemicals and Reagents

**Figure 1:**
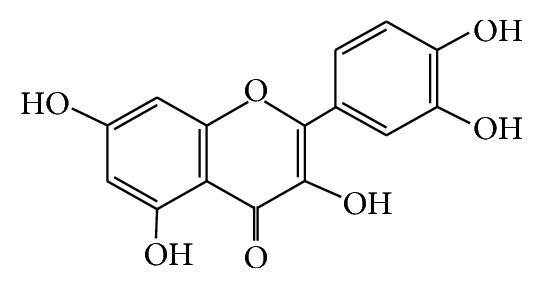
Quercetin structure formula.

**Figure 2:**
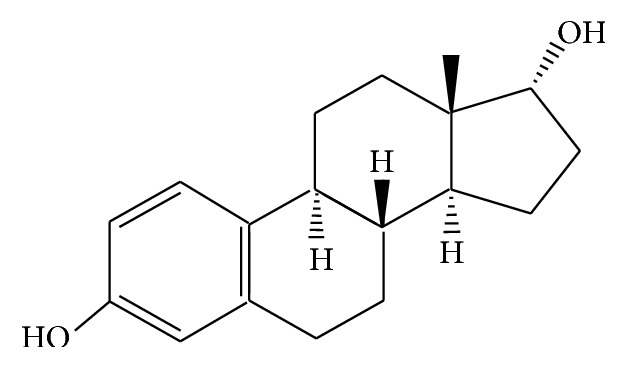
Estradiol structure formula.

Quercetin(4H-1-benzopyran-4-one,2-(3,4-dihydroxyphenyl)-3,5,7-trihydroxy-f0lavone) and C_15_H_10_O_7_ *·*2H_2_O (molecular weight 302.23, purity greater than 98%) were kind gifts from Swiss ALEXIS Biochemical Corporation. The structure of quercetin is shown in Figure 1, and its structure is similar with mammary estrogen 17b-estradiol (Figure 2), including a pair of hydroxy, the similar distance, and a phenol ring the latter one plays the decisive role in adsorbing on estrogen receptor. Gestrinone was purchased from Beijing ZiZhu Pharmaceutical (Beijing, China). Rat FSH and LH elisa Kits were purchased from USA Rapidbio Corporation. The mouse anti-ERa monoclonal antibody (68 kD), rabbit anti-ER*β* polyclonal antibody (55 kD), and mouse anti-PR antibody [PR-AT 4.14]—ChIP Grade monoclonal antibody (99 kD) were all purchased from ABCAM company.

### 2.3. Establishment of the Rat Model

According to the method of Jones [10], the following operation processes were conducted under a sterile condition. The rats were anesthetized with 3% pelltobarbitalum natricum before making a vertical incision on the abdomen. All the uteruses were removed and put into physiologic saline immediately. The endometrium was separated from the myometrium and cut into 0.5 × 0.5 cm pieces. The uterine segments were sutured onto the peritoneum close to blood vessels. The incision was closed and disinfected, and the animals were allowed to recover from anesthesia. Estradiol benzoate was subcutaneous injected for 3 days after the operation.

### 2.4. Group Administration and Drawing Materials

The volume (length × width × height) was measured by the electronic digital caliper. Rats with ectopic tissue volume larger than 20 mm^3^ were divided into 6 groups randomly by weight (Table 1).

After the administration of 21 days, the blood was drawn from abdominal aorta under anesthesia, and then the rats were executed and dissected. Blood was taken above 2.5 mL and centrifuged and the serum was taken and kept at −20°C. The hypothalamus, pituitary, eutopic and ectopic endometrium were removed, and tissues were partly fixed in 4% neutral paraformaldehyde and partly frozen in liquid nitrogen.

### 2.5. Enzyme-Linked Immunosorbent Assay (ELISA)

Samples should be placed into 2–8°C refrigerating for 5 days before being measured. We blended the serum upside down and performed the experiment according to the reagent kit's instructions (purchased from USA Rapidbio Corporation).

### 2.6. Immunohistochemistry

Tissues fixed in 4% neutral paraformaldehyde were flushed, dehydrated, waxed, and embedded in turn. Slides soaked in washing lotion overnight were rinsed with running water and dried. After being immersed in 100 pg/mL polylysine for 30 min at 37°C, slides were inserted into clean glass shelf overnight at 37°C. Cut 4 *μ*m serial thick sections with microtome, and 5 sections were selected for 1 h at 56°C and dried in 37°C overnight. Before staining, put the sections into 60°C warm case for 1 h. Then, sections were dewaxed in xylene and rehydrated, followed by microwaving in 10 mM sodium citrate buffer (PH 6.0) for antigen retrieval. Wash the sections with PBS (0.01 mmol/L, PH 7.4) for 5 min × 3. The following operations were conducted according to the reagent kit (immunohistochemistry S-P method, purchased from Maixin-Bio Corporation). The dilution ratio of ER*α*, ER*β*, and PR (abcam corporation) was 1 : 100.

### 2.7. Western Blotting

Total proteins in each group were extracted, and 10 *μ*L of protein in each group was loaded per lane, applied to a 10% polyacrylamide gel, and subjected to electrophoresis. The proteins were transferred to polyvinylidene difluoride membranes. After transfer, the membranes were blocked for 1 h in 5% BSA buffer, and then the membranes were incubated with ER*α*, ER*β*, and PR monoclonal antibodies in blocking buffer (diluted 1 : 800 with 5% BSA) for 90 min at 37°C. After being washed three times with PBST, the membranes were incubated with HRP-conjugated secondary antibodies (diluted 1 : 3000 with 5% BSA) for 60min at 37°C. The primary GAPDH antibody was diluted 1 : 10000 with 5% BSA. The membranes were washed three times with PBST, and photographs were taken immediately after color development using Gel Imaging System (bia-rad). The relative levels of protein were semiquantitatively determined using Image J analysis software. Set the internal concentration of each sample to a fixed value and determine the objective protein gray value of each sample and the gray value of GAPDH. The ratio of the objective protein gray value and the gray value of GAPDH represents the relative content of the objective protein.

### 2.8. Statistical Analysis

The data are presented as means ± SD. The statistical analysis of the results was done in SPSS 17.0 software (version 17.0 for Windows; SPSS, Chicago, IL, USA). The statistical significance among three or more groups was determined by one-way ANOVA analysis, and differences before and after treatment were evaluated by paired *t*-test. *P* < 0.05 was considered to be statistically significant.

## 3. Results

### 3.1. Effect of Quercetin on the Level of Serum FSH and LH

After the treatment, the levels of serum FSH and LH in each group were decreased (Table 2). The levels of serum FSH in quercetin high dose group, gestrinone group, and ovariectomized group are markedly decreased compared with model control group (*P* < 0.05), but there was no significant difference between quercetin high dose group and gestrinone group (*P* > 0.05). There was a pronounced decline on the level of serum LH in quercetin high and medium dose group, gestrinone group, and ovariectomized group compared with model control group (*P* < 0.05). Thus, quercetin and gestrinone can decrease the level of serum FSH and LH in our research.

### 3.2. Effect of Quercetin on the Expression of ER*α*, ER*β*, and PR in Hypothalamus

**Figure 3:**
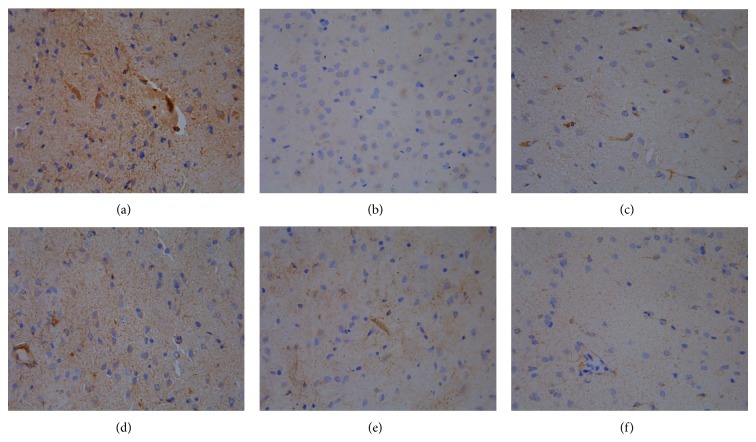
The expression of ER*α* in hypothalamus (DAB stain, ×400) ((a) model control group, (b) ovariectomized group, (c) gestrinone group, (d) quercetin low dose group, (e) quercetin medium dose group, and (f) quercetin high dose group).

**Figure 4:**
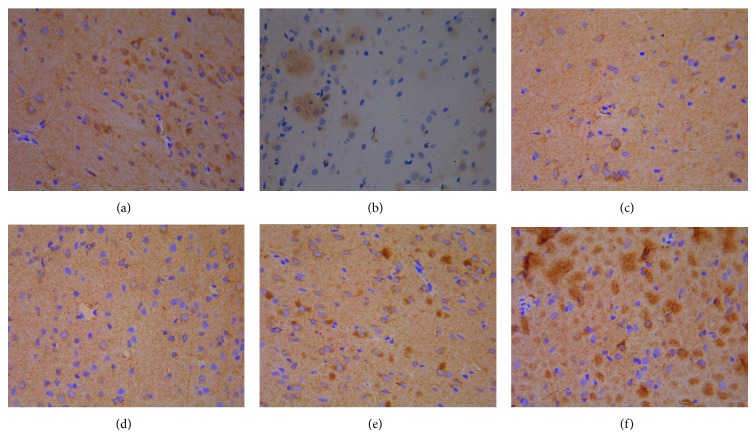
The expression of ER*β* in hypothalamus (DAB stain, ×400) ((a) model control group, (b) ovariectomized group, (c) gestrinone group, (d) quercetin low dose group, (e) quercetin medium dose group, and (f) quercetin high dose group).

**Figure 5:**
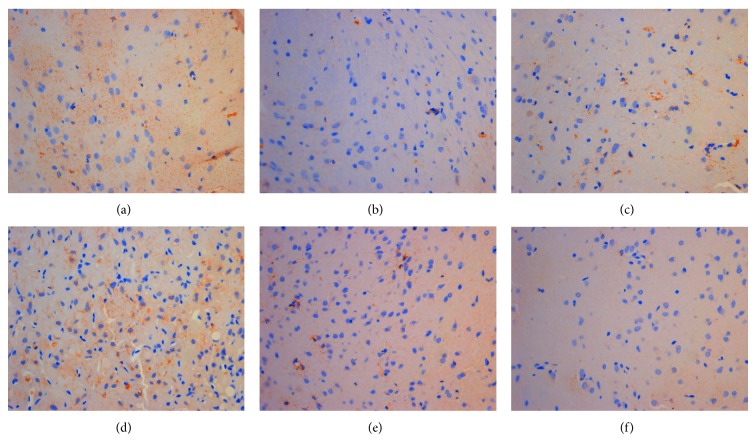
The expression of PR in hypothalamus (DAB stain, ×400) ((a) model control group, (b) ovariectomized group, (c) gestrinone group, (d) quercetin low dose group, (e) quercetin medium dose group, and (f) quercetin high dose group).

Results of immunohistochemistry showed that the integrated optical density (IOD) of ER*α* in model control group was significantly higher than ovariectomized group (*P* < 0.01). The IOD of ER*α* in quercetin low and medium dose group displayed no obvious difference in comparison with model control group (*P* > 0.05), and the IOD of ER*α* in quercetin high dose group was significantly reduced compared with model control group (*P* < 0.05). In addition to ovariectomized group, there was no obvious difference on the IOD of ER*β* in the other groups compared with model control group (*P* > 0.05). The IOD of PR was decreased in each group in comparison with model control group except for quercetin low dose group (*P* < 0.05) (Table 3 and Figures 3, 4, and 5).

### 3.3. Effect of Quercetin on the Expression of ER*α*, ER*β*, and PR in Eutopic Endometrium

**Figure 6:**
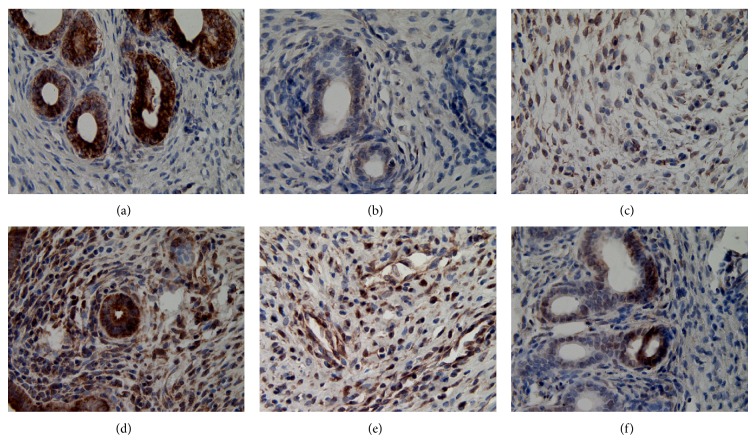
The expression of ER*α* in eutopic endometrium (DAB stain, ×400) ((a) model control group, (b) ovariectomized group, (c) gestrinone group, (d) quercetin low dose group, (e) quercetin medium dose group, and (f) quercetin high dose group).

**Figure 7:**
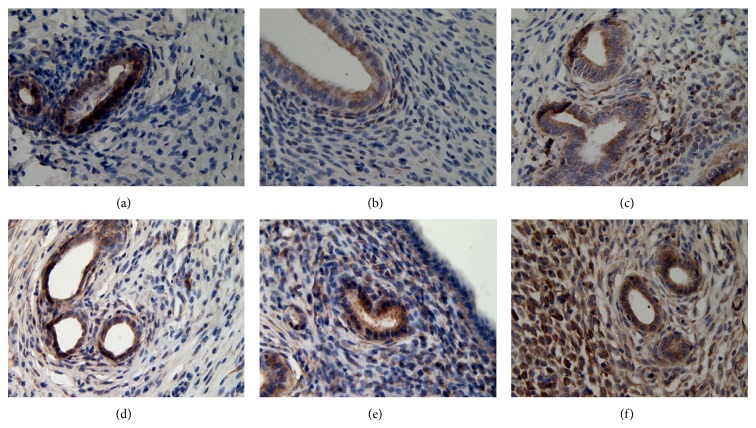
The expression of ER*β* in eutopic endometrium (DAB stain, ×400) ((a) model control group, (b) ovariectomized group, (c) gestrinone group, (d) quercetin low dose group (e) Quercetin medium dose group (f) Quercetin high dose group).

**Figure 8:**
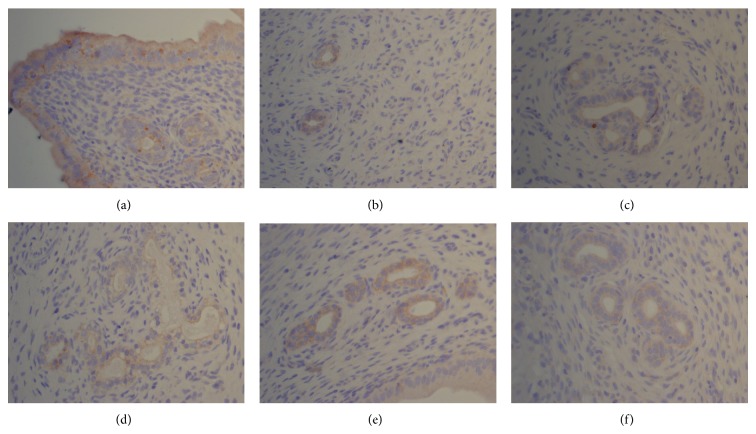
The expression of PR in eutopic endometrium (DAB stain, ×400) ((a) model control group, (b) ovariectomized group, (c) gestrinone group, (d) quercetin low dose group, (e) quercetin medium dose group, and (f) quercetin high dose group).

Results of immunohistochemistry showed that ER*α*, ER*β*, and PR expressed in uterine gland epithelial cells, luminal epithelial cells, and mesenchymal cells, with glandular epithelial cells expressed as strong. Due to the decline of ovarian hormone level, atrophy of the endometrium, and reduction of estrogen and progesterone receptor content, the IOD of ovariectomized group was significantly decreased in comparison with model control group (*P* < 0.01). Gestrinone can obviously increase the expression of ER*α* and PR (*P* < 0.05). Quercetin high dose group can downregulate the expression of ER*α* and PR and, meanwhile, upregulate the expression of ER*β* compared to model control group (*P* < 0.05) (Table 4 and Figures 6, 7, and 8).

### 3.4. Effect of Quercetin on the Expression of ER*α*, ER*β*, and PR in Ectopic Endometrium

**Figure 9:**
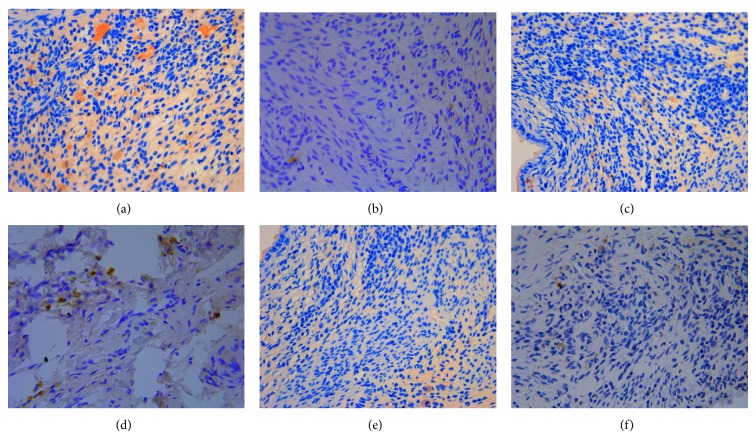
The expression of ER*α* in ectopic endometrium (DAB stain, ×400) ((a) model control group, (b) ovariectomized group, (c) gestrinone group, (d) quercetin low dose group, (e) quercetin medium dose group, and (f) quercetin high dose group).

**Figure 10:**
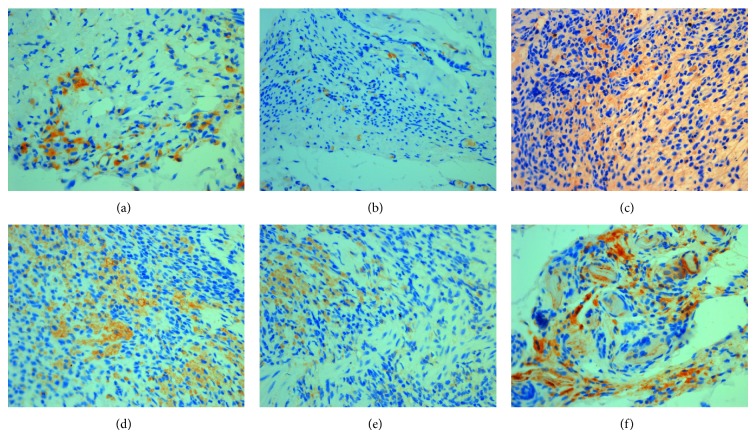
The expression of ER*β* in ectopic endometrium (DAB stain, ×400) ((a) model control group, (b) ovariectomized group, (c) gestrinone group, (d) quercetin low dose group, (e) quercetin medium dose group, and (f) quercetin high dose group).

**Figure 11:**
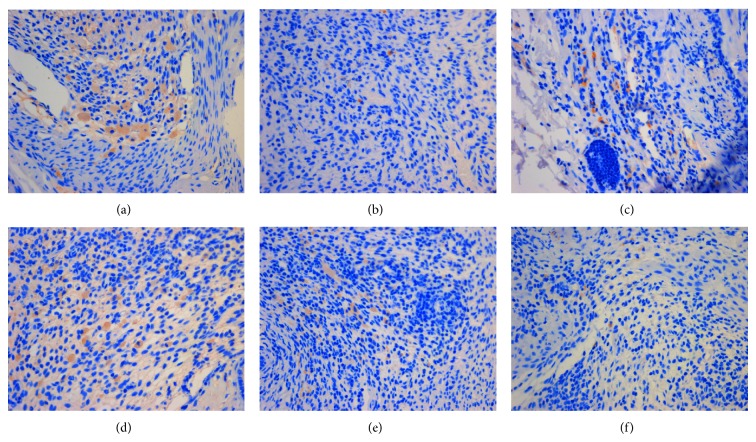
The expression of PR in ectopic endometrium (DAB stain, ×400) ((a) model control group, (b) ovariectomized group, (c) gestrinone group, (d) quercetin low dose group, (e) quercetin medium dose group, and (f) quercetin high dose group).

Results of immunohistochemistry showed that the expressions of ER*α*, ER*β*, and PR were detected mainly in glandular and luminal epithelial cells of ectopic endometrium and scattered in surrounding stromal cellular cytoplasm. After the treatment, the IOD of ER*α* in quercetin high dose group, gestrinone group, and ovariectomized group was reduced than model control group, and the difference had statistical significance (*P* < 0.05). There was a pronounced difference on the IOD of ER*β* between quercetin high dose group and model control group (*P* < 0.05). The IOD of PR was decreased in each group in comparison with model control group except for quercetin low dose group (*P* < 0.05) (Table 5 and Figures 9, 10, and 11).

### 3.5. Effect of Quercetin on the Expression and Quantity of ER*α*, ER*β*, and PR in Hypothalamus

**Figure 12:**
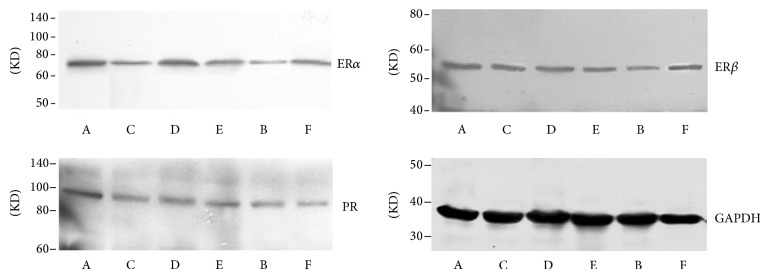
Effect of quercetin on the expression and quantity of ER*α*, ER*β*, and PR in hypothalamus, as assessed by western blotting.

Results of western blot showed that the expression of ER*α* and PR in hypothalamus in each groups was downregulated after the treatment, and there was obvious difference compared with model control group in addition to quercetin low dose group (*P* < 0.05). The expression of ER*β* in quercetin low dose group, ovariectomized group and gestrinone group was significantly decreased in comparison with model control group (*P* < 0.05). The ratio of ER*α*/ER*β* in model control group was higher than quercetin high dose group (Table 6 and Figure 12).

### 3.6. Effect of Quercetin on the Expression and Quantity of ER*α*, ER*β*, and PR in Pituitary

**Figure 13:**
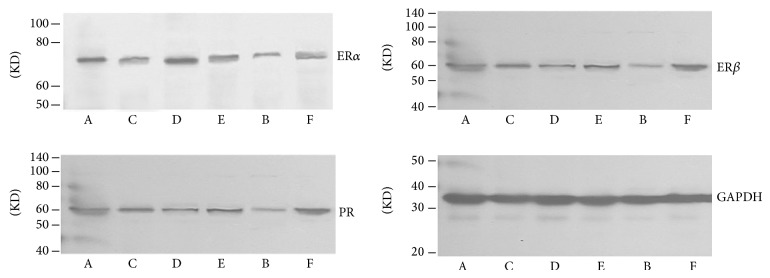
Effect of quercetin on the expression and quantity of ER*α*, ER*β*, and PR in pituitary, as assessed by western blotting.

Results of western blot showed that the expression of ER*α* in pituitary in quercetin high and medium dose group, ovariectomized group, and gestrinone group was downregulated after the treatment, and there was obvious difference compared with model control group (*P* < 0.01). In addition to ovariectomized group, the expression of ER*β* between other groups and model control group showed no statistical significance (*P* > 0.05). There was a pronounced difference on the expression of PR in ovariectomized group and gestrinone group compared with model control group (*P* < 0.05). The ratio of ER*α*/ER*β* in quercetin high dose group and ovariectomized group was significantly lower than model control group (*P* < 0.01) (Table 7 and Figure 13).

### 3.7. Effect of Quercetin on the Expression and Quantity of ER*α*, ER*β*, and PR in Eutopic Endometrium

**Figure 14:**
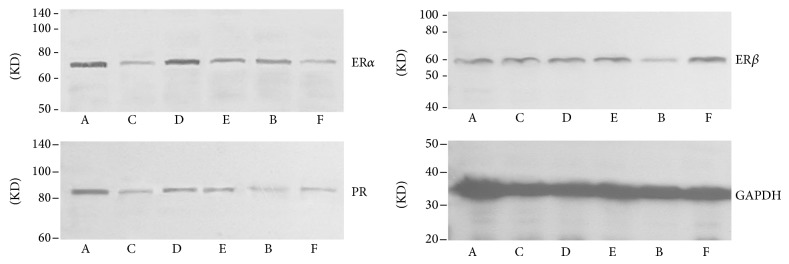
Effect of quercetin on the expression and quantity of ER*α*, ER*β*, and PR in eutopic endometrium, as assessed by western blotting.

Results of western blot showed that there was pronounced difference on the expression of ER*α* in eutopic endometrium in each group compared with model control group in addition to quercetin low dose group (*P* < 0.01). The expression of ER*β* was upregulated except for ovariectomized group, and there was significant difference between quercetin high dose group and model control group (*P* < 0.01). The expression of PR in each groups was obvious downregulated in comparison with model control group (*P* < 0.01). In addition to quercetin low dose group, the ratio of ER*α*/ER*β* in other groups was decreased, and the difference had statistical significance (*P* < 0.01) (Table 8 and Figure 14).

## 4. Discussion

Endometriosis is a hormone dependent disease; it relays on both estrogen and progesterone. Increased estrogen in local tissue is the key factor for ectopic endometrial cells to successful implantation, and there are scholars who believe that estrogen receptor gene polymorphism is closely related to the occurrence and development of EMs [11]. The past researches suggested that estrogen took effect only through the single and classic ER*α*, and with the discovery of ER*β*, more evidence indicates that the pathophysiology mechanism of estrogen to target cells is regulated by ER*α* and ER*β* [12]. ER alpha and beta differ in tissue distribution. Studies found that ER*β* expressed more widely than ER*α*, particularly in non-reproductive tissue, such as brain, pituitary and vascular system, and there was low expression or no expression in ovary. On the contrary, ER*α* expressed more obvious in reproductive system [13]. So it can be concluded that ER*α* mainly mediated the function of estrogen in reproductive tissues, while the effect of estrogen in other tissues such as brain was mediated by ER*β*. From the physiological aspect, in addition to the role in the reproductive system, ER*β* plays a major role in proliferation and differentiation of neurons, astrocytes, and female reproductive cells [14]. In the brain, estrogen receptor ER*α* and ER*β* mediated the persistent regulation of estrogen to nerve cells in the target gene transcription, and cells in anterior and middle hypophysis are targets of estrogen action. ER*α* express highly in most cells in the pituitary, and the expression of ER*β* is in medium level [15]. ER*α* mediates the negative feedback effect of estrogen on the brain [16], while ER*β* may be involved in the regulation of higher brain functions such as neurodegenerative diseases of learning and memory [17]. In addition, estrogen can induce the expression of PR, and it is closely related to EMs. Estrogen can induce the estrous cycle in ovariectomized rats and simulate the endocrine environment before the normal estrous, and these are realized by upregulating the expression of PR and increasing the release pathway of LHRH [18]. This suggests that the relationship between the specific expression of PR on the HPGA axis and reproductive function is important.

Progesterone is a commonly used drug for the treatment of endometriosis in recent years. The pain symptoms in about 3/4 of patients with EMs can be effectively controlled by progesterone, and the effect is not inferior to other traditional medicine. It is generally believed that the action mechanism of progesterone had two aspects. On one hand, it can directly act on the endometrium, making it into decidua and then shrink. On the other hand, it can directly affect the pituitary gland, affecting the secretion of follicle-stimulating hormone (FSH) and the ratio of FSH and luteinizing hormone (LH). Our experiment selected gestrinone as the positive control drug, which is one of the main drugs for the clinical treatment of EMs. It has antiprogesterone and antiestrogen effect, and it also has moderate antigonadotropic effect. It can inhibit the release of gonadotropin (FSH and LH) and cause the inhibition of ovarian secretion function, and the levels of progesterone and estrogen in the blood are decreased. In addition, it can also combine with androgen protein in the blood and increases the level of free testosterone in the body, directly inhibiting the growth of endometrium and making it atrophy and absorption [19]. Literatures reported that the levels of ER and PR and the ratio of ER/PR in ectopic endometrium of endometriosis patients were significantly lower than those in the endometrium of the control group, and they often sustained proliferative changes. This suggests that changes of hormone receptor content in ectopic endometrium may affect the local estrogen metabolism and lead to the sustained growth in ectopic endometrium [20]. In our experiment, the results of immunohistochemistry confirmed that the expressions of ER*α* and ER*β* and PR in ectopic endometrium were significantly lower than in eutopic endometrium, which was consistent with the reported literatures.

Quercetin is one of flavones in phytoestrogen. High-mass matrix-assisted laser desorption/ionization mass spectrometry (MALDI-MS) combined with chemical cross-linking displays that quercetin has a stronger binding affinity for hERa LBD (0.01%) [21]. So quercetin has the same features with other flavonoid phytoestrogens, which is the dose-dependent effect on the disease. When the body has enough estrogen levels, that is, women in the reproductive period of estrogen in the body at a relatively high level, phytoestrogen plays antiestrogen activity role. Our study found that high dose quercetin can downregulate the level of the serum FSH and LH in rats, and this may be the effect of quercetin on the HPGA axis. This is related to the inhibition of increased LH and FSH level before ovulation and causes the decrease of plasma concentrations of estradiol and thereby inhibits the growth of ectopic endometrium. In addition, studies showed that quercetin can downregulate the expression of catechol-O-methyltransferase (COMT) and inhibited its activity by estrogen receptor, and it can reduce the influence of estrogen metabolites on breast cancer [22]. Quercetin can also directly upregulate the expression of ER*β* in breast cancer T47D-p cells and affect the ratio of ER*α*/ER*β* and eventually inhibit cell growth and proliferation [23]^.^ Therefore, the regulation of quercetin on estrogen metabolism makes it become the research focus on the treatment of EMs in recent years.

Results of western blot in our study showed that quercetin can significantly downregulate the expression of ER*α* and PR in endometrium and at the same time increase the expression of ER*β*, resulting in the decrease of ER*α*/ER*β*, thus changing the ectopic planted ability of endometrium in EMs rats. Quercetin only inhibited the expression of ER*α* in the uterine but had no significant effect on ER*β*, so it can be understood that the direct effect of quercetin on the uterus is mainly realized by the adjustment of ER*α* rather than ER*β*. In reproductive system such as the uterus, ER*α* is the major receptor subtype which estrogen worked and combined with. Furthermore, ER*β* had a clear effect on the inflammatory response of ovarian, cardiovascular, brain diseases, arthrophlogosis, endometriosis, and so on [20]. So it is suggested that quercetin may have no significant improving effect on the inflammatory response of endometrium mediated by ER*β*.

In the hypothalamus, we found that quercetin can decrease the expression of ER*α* and the ratio of ER*α*/ER*β* and had a significant downregulation on the expression of PR. In the pituitary, quercetin can decrease the expression of ER*α* but increase the expression of ER*β*, but the effect is not very significant, ultimately leading to the obvious decrease on the ratio of ER*α*/ER*β*. This suggested that the action mechanism of quercetin in the hypothalamus is directly reducing the expression of ER*α* and then inhibiting the expression of PR, the induction of PR on LH, the sensibility of ER*α* on estrogen feedback regulation, a series of signal pathway effect caused by ER*α* and PR, and the promoting transcription of estrogen and progesterone on the downstream gene.

According to the above results, the mechanism of quercetin inhibiting the growth of ectopic endometrium in rat model is that quercetin can be combined with the receptor ER*α* of the HPGA axis, feedback inhibits the release of GnRH, and exerts its antigonadotropic activity to inhibit the secretion of FSH and LH and to reduce the level of serum E_2_, causing the deficiency of hormone supplement in ectopic endometrium, thus reducing the height of endometrial epithelial layer and the number of ectopic endometrial glands, eventually leading to the atrophy of ectopic endometrial epithelial. However, in summary, ER and PR distributed in the reproductive system and the central nervous system play different roles, and how quercetin regulates their expression and changes the downstream pathways related with ER and PR is worthy of future study.

## Figures and Tables

**Table 1:** Group administration and drawing materials.

Group	Drug	*N*	Dosage
Model control group	0.5% CMC	7	5 mL/(kg*·*d) × 21 d
Ovariectomized group	0.5% CMC	6	5 mL/(kg*·*d) × 21 d
Gestrinone group	Gestrinone	7	5 mg/(kg*·*2 d) × 10 d
Quercetin low dose group	Quercetin	7	60 mg/(kg*·*d) × 21 d
Quercetin medium dose group	Quercetin	7	150 mg/(kg*·*d) × 21 d
Quercetin high dose group	Quercetin	7	375 mg/(kg*·*d) × 21 d

**Table 2:** Posttreatment levels of serum FSH and LH in each group $\left(\overset{-}{x}\pm s\right)$.

Group	*N*	FSH (IU/L)	LH (IU/L)
Model control group	7	4.56 ± 0.45	12.45 ± 2.51
Ovariectomized group	6	2.32 ± 0.54∗	6.34 ± 0.56∗∗
Gestrinone group	7	3.22 ± 0.17∗	8.48 ± 1.55∗∗
Quercetin low dose group	7	3.53 ± 0.58	11.32 ± 1.58
Quercetin medium dose group	7	3.42 ± 0.49	10.07 ± 1.56∗
Quercetin high dose group	7	2.73 ± 0.19∗	7.88 ± 1.11∗∗

∗Express *P* < 0.05, ∗∗express *P* < 0.01, compared with the model control group.

**Table 3:** Effect of quercetin on the expression of ER*α*, ER*β*, and PR in hypothalamus $\left(\overset{-}{x}\pm s\right)$.

Group	*N*	ER*α*	ER*β*	PR
Model control group	7	5.20*E*4 ± 2.19*E*4	1.87*E*5 ± 8.95*E*4	3.06*E*4 ± 1.44*E*4
Ovariectomized group	6	1.13*E*4 ± 5.51*E*3∗∗	8.42*E*4 ± 3.93*E*4∗∗	1.02*E*4 ± 9.52*E*3∗
Gestrinone group	7	3.15*E*4 ± 9.36*E*3∗	1.14*E*5 ± 2.01*E*4	1.04*E*4 ± 2.76*E*3∗
Quercetin low dose group	7	6.37*E*4 ± 7.73*E*3	1.54*E*5 ± 3.55*E*4	2.15*E*4 ± 1.16*E*4
Quercetin medium dose group	7	3.55*E*4 ± 6.88*E*3	1.56*E*5 ± 3.42*E*4	1.21*E*4 ± 4.71*E*3∗
Quercetin high dose group	7	2.69*E*4 ± 8.76*E*3∗	1.99*E*5 ± 5.60*E*4	1.12*E*4 ± 2.14*E*3∗

∗Express *P* < 0.05, ∗∗express *P* < 0.01, compared with the model control group.

**Table 4:** Effect of quercetin on the expression of ER*α*, ER*β*, and PR in eutopic endometrium $\left(\overset{-}{x}\pm s\right)$.

Group	*N*	ER*α*	ER*β*	PR
Model control group	7	3.90*E*5 ± 9.19*E*4	2.18*E*5 ± 1.74*E*4	1.25*E*4 ± 6.29*E*3
Ovariectomized group	6	9.89*E*4 ± 1.34*E*4∗∗	8.67*E*4 ± 1.59*E*4∗∗	1.33*E*3 ± 1.08*E*3∗∗
Gestrinone group	7	1.34*E*5 ± 2.37*E*4∗∗	2.22*E*5 ± 4.14*E*4	3.43*E*3 ± 1.76*E*3∗
Quercetin low dose group	7	3.20*E*5 ± 1.21*E*4	1.91*E*5 ± 5.17*E*4	1.04*E*4 ± 5.81*E*3
Quercetin medium dose group	7	2.15*E*5 ± 8.10*E*3∗	2.16*E*5 ± 6.76*E*4	7.10*E*3 ± 5.77*E*3
Quercetin high dose group	7	2.29*E*5 ± 8.58*E*4∗	3.25*E*5 ± 5.83*E*4∗	4.38*E*3 ± 2.52*E*3∗

∗Express *P* < 0.05, ∗∗express *P* < 0.01, compared with the model control group.

**Table 5:** Effect of quercetin on the expression of ER*α*, ER*β*, and PR in ectopic endometrium $\left(\overset{-}{x}\pm s\right)$.

Group	*N*	ER*α*	ER*β*	PR
Model control group	7	4.49*E*4 ± 2.22*E*4	1.83*E*4 ± 1.41*E*4	1.07*E*4 ± 1.63*E*3
Ovariectomized group	6	1.90*E*4 ± 4.94*E*3∗	0.77*E*4 ± 2.07*E*3	3.37*E*3 ± 4.99*E*2∗∗
Gestrinone group	7	1.96*E*4 ± 6.11*E*3∗	1.32*E*4 ± 7.62*E*3	5.12*E*3 ± 2.36*E*3∗
Quercetin low dose group	7	4.52*E*4 ± 1.80*E*4	1.46*E*4 ± 1.08*E*4	7.64*E*3 ± 4.20*E*3
Quercetin medium dose group	7	2.63*E*4 ± 1.48*E*4	1.49*E*4 ± 7.62*E*3	6.18*E*3 ± 3.10*E*3∗
Quercetin high dose group	7	2.01*E*4 ± 4.35*E*3∗	3.27*E*4 ± 1.67*E*4∗	3.85*E*3 ± 7.06*E*2∗∗

∗Express *P* < 0.05, ∗∗express *P* < 0.01, compared with the model control group.

**Table 6:** Effect of quercetin on the expression and quantity of ER*α*, ER*β*, and PR in hypothalamus $\left(\overset{-}{x}\pm s\right)$.

Group	*N*	ER*α*	ER*β*	PR	ER*α*/ER*β*
Model control group	7	4.09*E* − 01 ± 4.85*E* − 02	2.91*E* − 01 ± 4.04*E* − 02	2.99*E* − 01 ± 3.55*E* − 02	1.44 ± 0.32
Ovariectomized group	6	1.33*E* − 01 ± 5.18*E* − 02^**^	1.30*E* − 01 ± 1.16*E* − 02^**^	1.51*E* − 01 ± 1.79*E* − 02^**^	1.05 ± 0.49
Gestrinone group	7	2.38*E* − 01 ± 4.61*E* − 02^**^	2.20*E* − 01 ± 4.49*E* − 02^**^	1.59*E* − 01 ± 3.77*E* − 02^**^	1.12 ± 0.27
Quercetin low dose group	7	4.07*E* − 01 ± 3.08*E* − 02	2.36*E* − 01 ± 4.77*E* − 02^*^	2.63*E* − 01 ± 5.12*E* − 02	1.79 ± 0.43
Quercetin medium dose group	7	3.27*E* − 01 ± 7.61*E* − 01^**^	2.65*E* − 01 ± 5.28*E* − 02	2.32*E* − 01 ± 5.47*E* − 02^*^	1.30 ± 0.45
Quercetin high dose group	7	2.10*E* − 01 ± 4.94*E* − 01^**^	2.72*E* − 01 ± 3.94*E* − 02	1.84*E* − 01 ± 7.42*E* − 02^**^	0.78 ± 0.20^**^

∗Express *P* < 0.05, ∗∗express *P* < 0.01, compared with the model control group.

**Table 7:** Effect of quercetin on the expression and quantity of ER*α*, ER*β*, and PR in pituitary $\left(\overset{-}{x}\pm s\right)$.

Group	*N*	ER*α*	ER*β*	PR	ER*α*/ER*β*
Model control group	7	4.17*E* − 01 ± 7.87*E* − 02	3.64*E* − 01 ± 4.46*E* − 02	2.68*E* − 01 ± 4.89*E* − 02	1.15 ± 0.21
Ovariectomized group	6	1.23*E* − 01 ± 1.47*E* − 02^**^	2.12*E* − 01 ± 4.97*E* − 02^**^	1.07*E* − 01 ± 4.26*E* − 02^**^	0.61 ± 0.17^**^
Gestrinone group	7	3.04*E* − 01 ± 7.52*E* − 02^**^	3.95*E* − 01 ± 1.22*E* − 01	1.67*E* − 01 ± 6.74*E* − 02^*^	0.81 ± 0.25
Quercetin low dose group	7	3.99*E* − 01 ± 8.42*E* − 02	3.98*E* − 01 ± 2.38*E* − 01	2.54*E* − 01 ± 1.03*E* − 01	1.25 ± 0.57
Quercetin medium dose group	7	2.75*E* − 01 ± 5.96*E* − 02^**^	4.24*E* − 01 ± 1.71*E* − 01	2.71*E* − 01 ± 1.13*E* − 01	0.72 ± 0.29
Quercetin high dose group	7	2.25*E* − 01 ± 5.68*E* − 02^**^	5.13*E* − 01 ± 2.04*E* − 01	2.43*E* − 01 ± 6.90*E* − 02	0.48 ± 0.17^**^

∗Express *P* < 0.05, ∗∗express *P* < 0.01, compared with the model control group.

**Table 8:** Effect of quercetin on the expression and quantity of ER*α*, ER*β*, and PR in eutopic endometrium $\left(\overset{-}{x}\pm s\right)$.

Group	*N*	ER*α*	ER*β*	PR	ER*α*/ER*β*
Model control group	7	3..40*E* − 01 ± 9.53*E* − 02	3.36*E* − 01 ± 4.54*E* − 02	2.74*E* − 01 ± 3.31*E* − 02	1.01 ± 0.23
Ovariectomized group	6	8.19*E* − 02 ± 2.38*E* − 02^**^	1.93*E* − 01 ± 5.12*E* − 02^**^	7.42*E* − 02 ± 2.03*E* − 02^**^	0.43 ± 0.13^**^
Gestrinone group	7	1.85*E* − 01 ± 4.53*E* − 02^**^	3.60*E* − 01 ± 9.60*E* − 02	1.01*E* − 01 ± 1.67*E* − 02^**^	0.54 ± 0.18^**^
Quercetin low dose group	7	2.80*E* − 01 ± 6.11*E* − 02	3.19*E* − 01 ± 4.46*E* − 02	1.71*E* − 01 ± 4.05*E* − 02^**^	0.87 ± 0.10
Quercetin medium dose group	7	2.03*E* − 01 ± 4.90*E* − 02^**^	3.57*E* − 01 ± 3.42*E* − 02	1.62*E* − 01 ± 3.68*E* − 02^**^	0.57 ± 0.13^**^
Quercetin high dose group	7	1.79*E* − 01 ± 5.19*E* − 02^**^	4.52*E* − 01 ± 7.96*E* − 02^**^	1.27*E* − 01 ± 2.08*E* − 02^**^	0.40 ± 0.12^**^

∗Express *P* < 0.05, ∗∗express *P* < 0.01, compared with the model control group.
